# Fetal lung C4BPA induces p100 processing in human placenta

**DOI:** 10.1038/s41598-019-42078-0

**Published:** 2019-04-02

**Authors:** Mayra Cruz Ithier, Nataliya Parobchak, Stacy Yadava, Jerry Cheng, Bingbing Wang, Todd Rosen

**Affiliations:** 1 Department of Obstetrics and Gynecology, Division of Maternal-Fetal Medicine, Rutgers Robert Wood Johnson Medical School, New Brunswick, NJ 08901 USA; 2 The Cardiovascular Institute of New Jersey, Rutgers Robert Wood Johnson Medical School, New Brunswick, NJ 08901 USA

## Abstract

The non-canonical NF-κB signaling may be a central integrator of a placental clock that governs the length of human pregnancy. We sought to identify fetal signals that could activate this NF-κB pathway in the placenta, and in turn, contribute to the onset of labor. Proteomics analysis of exosomes purified from fetal cord arterial blood revealed a total of 328 proteins, among which 48 were more significantly abundant (p < 0.01) in samples from women who delivered following elective Cesarean-section at term (39 to 40 weeks of estimated gestational age, EGA) compared to those who had elective Cesarean deliveries near term (35 to 36 weeks of EGA). Computational, crystal structural, and gene functional analyses showed that one of these 48 proteins, C4BPA, binds to CD40 of placental villous trophoblast to activate p100 processing to p52, and in turn, pro-labor genes. These results suggest that fetal C4BPA-induced activation of non-canonical NF-κB in human placenta may play a critical role in processes of term or preterm labor.

## Introduction

Human parturition is a precisely coordinated process resulting from activation of a series of endocrine and immune responses, and a premature and sustained activation of these responses is hypothesized to lead to cases of preterm birth. Most therapeutic approaches to prevent preterm birth are currently ineffective^1,2^, and our limited success in preventing preterm birth suggests an incomplete understanding of the biological clock that governs the length of pregnancy and triggers parturition.

We have been studying the molecular mechanisms underlying glucocorticoid-mediated surge of COX-2 and CRH with use of primary cultures of cytotrophoblast (CTB) and have developed a plausible model that explains how the placenta and fetus may interact to contribute to initiation of labor. Briefly, we have ascribed the non-canonical NF-κB (RelB-p100/p52) pathway and associated epigenetic modifications a central regulatory role in regulation of the placental clock^3,4^. Placental stress characterized by glucocorticoid excess from the growing fetus drives RelB/p100 (NF-κB2) production^5^. However, the mechanisms triggering processing of NF-κB2 precursor form p100 to active p52 have not been elucidated.

Recently, Gao and colleagues found that signals from the mature fetal lung caused involution of the corpus luteum to initiate labor in an Src-1/Src-2 knockout mouse^6^. There are numerous important differences between the mechanisms that govern human and murine labor, but we sought analogous signals produced by the fetus that may contribute to the onset of parturition in humans.

Recent advances in understanding cell-to-cell communication have witnessed a significant shift with the recognition of the role of exosomes in intracellular signaling^7^. Over the past few years, evidence has begun to accumulate that the extracellular vesicles such as exosomes contain cell-specific collections of proteins, lipids, and genetic material that are transported to target cells where they are capable of modifying function and physiology.

In the present study, we tested the hypothesis that the fetus influences placental function by packaging signaling proteins in exosomes, and that these signals could be a trigger that initiates parturition. By performing proteomics analysis on purified exosomes of fetal cord arterial blood, we identified a total of 328 proteins including C4BPA, a gene controlling the canonical pathway of complement activation, in exosomes isolated from umbilical artery blood. Co-immunoprecipitation and functional assays suggest that C4BPA originating from the fetal lung served as a potent trigger for p100 processing. These results suggest that fetal C4BPA-induced activation of non-canonical NF-κB in human placenta may play a critical role in initiation of human parturition.

## Results

### Proteomic profiling of fetal cord blood exosomes

We hypothesized that the signals that trigger labor should occur late in pregnancy and umbilical artery blood presumably contains the greatest concentration of signaling molecules produced directly by the fetus, having just passed through the fetal systemic circulation. We further hypothesized that the arterial exosomes of fetal origin should be uptaken by the placenta after passing the placental circulation, which in turn should be remarkably reduced in umbilical vein blood.

**Figure 1:**
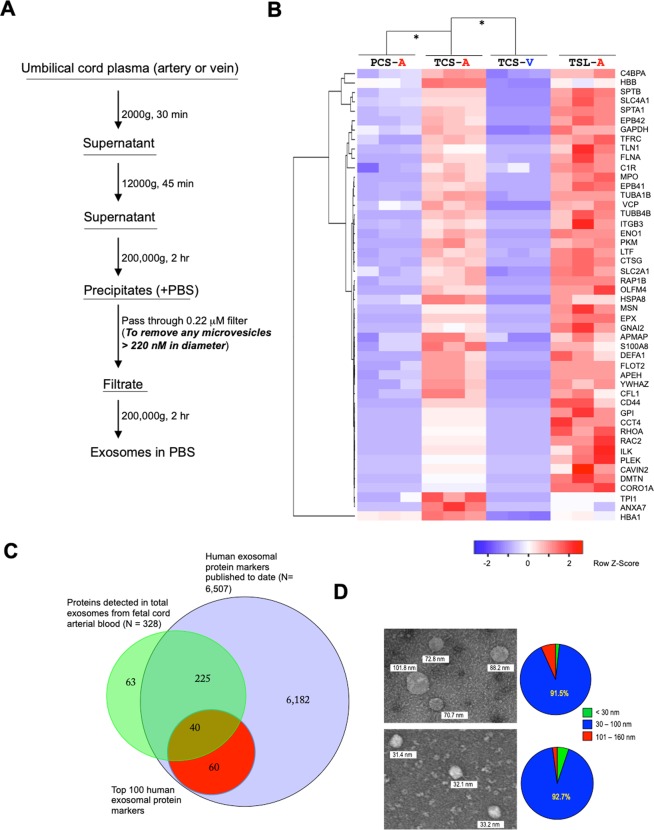
Proteomics analysis of fetal cord blood exosomes. (**A**) Isolation procedure of fetal cord blood exosomes. (**B**) The heatmap showing select proteins with significant difference in normalized abundance in fetal exosomes among groups as indicated (*p < 0.01). PCS-A, arterial blood exosomes isolated from patients of 35 to 36 weeks of EGA following C-section; TCS-A, arterial blood exosomes isolated from patients of 39 to 40 weeks of EGA following C-section; TCS-V, vein blood exosomes isolated from the same patients of 39 to 40 weeks of EGA following C-section; TSL-A, arterial blood exosomes isolated from patients of 39 to 40 weeks of EGA following spontaneous labor and vaginal delivery. The complete proteomics data is shown in Supplementary Table 1. (**C**) Venn diagram showing the number of exosomal proteins identified in this study, which were overlapped with those published to date^11^. Red circle indicates top 100 exosomal protein markers. (**D**) Imaging of exosomes isolated from the cord arterial blood of the groups as indicated by transmission electron microscope (left panels). For each group, the percentage of microvesicles based on the sizes as indicated was calculated from 10 random fields (right panels). Original magnification, X 45,000.

Cord blood exosomes were isolated as described in Fig. 1A^8^. We first performed an *in vitro* uptake assay and found that cord arterial exosomes were effectively uptaken by term human CTB (Supplementary Fig. 1). Next, we performed proteomics analysis on the exosomes extracted from umbilical artery blood immediately following delivery of human pregnancies from 3 groups: (1) Patients receiving elective C-section with 35 to 36 weeks of EGA (PCS-A); (2) Patients following C-section at term with 39 to 40 weeks of EGA (TCS-A); and (3) Patients following spontaneous labor and vaginal delivery with EGA between 39 and 40 weeks EGA (TSL-A). We also collected cord vein blood specimens from the same patients of TCS (TCS-V). We identified a total of 328 polypeptides in the arterial cord blood specimens (Supplementary Table 1) with 48 proteins more abundant in the TCS-A compared to the PCS-A group (p ≤ 0.01) (Fig. 1B). Proteomics were also performed on exosomes isolated TCS-V and compared to TCS-A specimens. All 48 of the proteins of interest seemed to be present in higher quantity in the arterial specimens compared to the matched venous specimens from the same subjects. These results suggest that these proteins are secreted in the form of exosomes by the fetus and enter the circulation to exert their biological effects on the placenta.

The stress induced by labor could be a driver for the proteins differentially secreted by the fetus between early and term pregnancy. In the placenta, catalase (CAT), a key antioxidant enzyme in the body defense against oxidative stress, and von Willebrand factor (VWF), a glycoprotein involved in hemostasis increase with labor duration and its associated stress^9^. In addition, multiple heat shock proteins (HSP) can protect cells against different stresses^10^. We did not find any significant differences in the abundance of CAT, VWF, or HSPA8 in exosomes derived from the umbilical artery between the TCS-A and TSL-A (Supplementary Table 1). Furthermore, of 48 proteins aforementioned, only 4 including SPTA1, C1R, CTSG, and CORO1A showed an increase in the TSL-A compared to the TCS-A group (p < 0.01). These results suggest that labor-associated stress has minimal effects on the contents of fetal exosomal proteins and that difference in exosomal contents between early and term pregnancy is indeed associated with fetal maturation.

In order to determine the exosomes are biologically *bona fide*, we compared the composition of exosomal protein markers detected in this study to those previously published. To date, there are a total of 6,507 protein identified within the exosomes secreted by a variety of types of human tissues^11^. Here we found that 265 of 328 exosomal proteins identified in the current study have been previously identified as exosomal protein markers (Fig. 1C). The high homogeneity and purity of the exosomes isolated were further supported by transmission electron microscope (TEM) imaging (Fig. 1D). Furthermore, these results suggest that the remaining 63 proteins could serve as novel exosomal protein markers.

### Exosomal C4BPA originates from fetal lung

Because exosomes function by communicating signal molecules contained inside to target cells, we first used STRING, a program for functional protein association networks^12^ to predict protein-protein interaction.

By individually searching these 48 proteins as described above against the STRING, we found C4BPA, a gene controlling the canonical pathway of complement activation, to be of particular interest. C4BPA is the only protein predicted to interact with CD40 (Supplementary Fig. 2), one of four TNF receptor superfamily members which can trigger activation of non-canonical NF-κB signaling^13^. Previously, C4BPA has been shown to activate B-lymphocytes by binding CD40^14^. Interestingly, RNA-sequencing (RNA-seq) of mid-trimester and term CTB demonstrated that only the TNF receptor superfamily members CD40 and the lymphotoxin β−receptor (LTβR), but not receptor activator for nuclear factor κB (RANK) and B-cell-activating factor (BAFFR), were robustly expressed in the human placenta (Supplementary Fig. 3).

**Figure 2:**
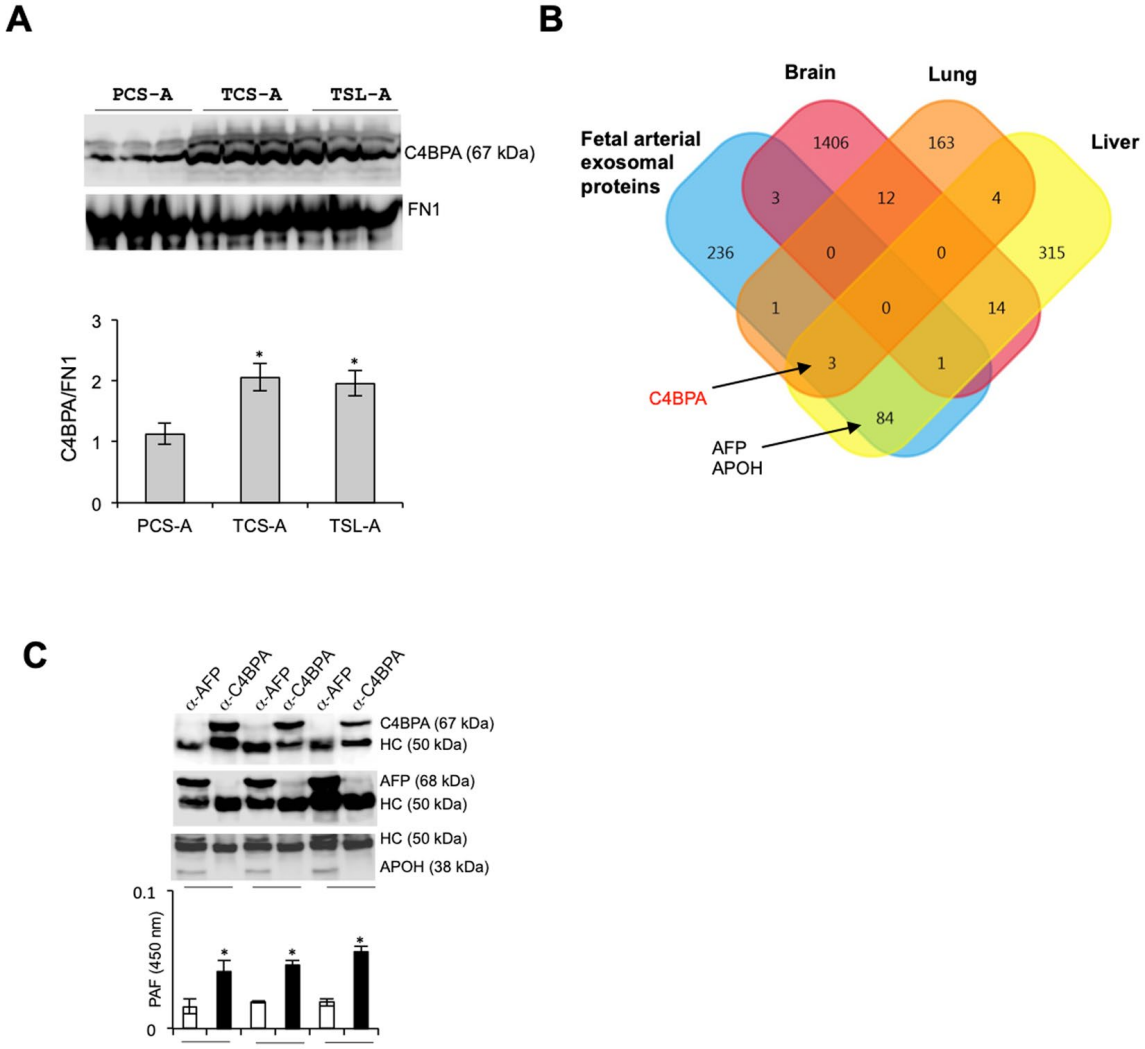
C4BPA is contained in fetal lung exosomes. (**A**) Validation of differential abundance of exosomal C4BPA among groups as indicated by Western blot analysis (top panels). C4BPA abundance was normalized to FN1 and quantified with use of ImageJ program^33^. Student t-test was used compare either results of the TCS-A or TSL-A with those of the PCS-A. *p < 0.01 (N = 3 individual samples). (**B**) Venn diagram showing number of tissue-specific proteins that were obtained from the Human Protein Atlas^15^, indicating that C4BPA, which was identified in fetal exosomes, originates from fetal liver or lung, whereas AFP and APOH are found exclusively in liver. (**C**) Anti-C4BPA or -AFP antibody was used in immunoprecipitation (IP) assay of fetal exosomes prepared from the samples of 3 individual TCS-A and IPs were analyzed by means of Western blot for the listed proteins or by ELISA for identifying PAF. The bars represent average of A450 values and standard deviation from triplicates by the same IPs. Student t-test was used to compare values of A450 between α-AFP and α-C4BPA within the same sample. *p < 0.01.

Next, we used Western blot analysis to further confirm increased abundance of C4BPA in arterial blood specimens of either the TCS or TSL compared to those of PCS (Fig. 2A, Supplementary Fig. 4). C4BPA can be produced in either human liver or lung (Fig. 2B, Supplementary Fig. 5)^15^. We selectively captured exosomes by immunoprecipitation and then assayed for other associated proteins to determine whether exosomal C4BPA in fetal cord blood originated from the liver, lung or both organs. We did not identify C4BPA in exosomes captured with antibody against α-fetoprotein (AFP), a protein produced exclusively in the liver during fetal life^16^. We did capture apolipoprotein H (APOH), another protein mainly produced in the liver and gallbladder, in the AFP-positive exosomes. Conversely, we were able to detect platelet-activating factor, a proinflammatory glycerophospholipid secreted by mature fetal lung^6^, but not APOH in the IPs with C4BPA antibody (Fig. 2C, Supplementary Fig. 6).

These data strongly suggest that C4BPA is produced predominantly in fetal lung during pregnancy, and the liver becomes the major site for production of C4BPA in adult life.

### Interaction of heptameric C4BPA and CD40

**Figure 3:**
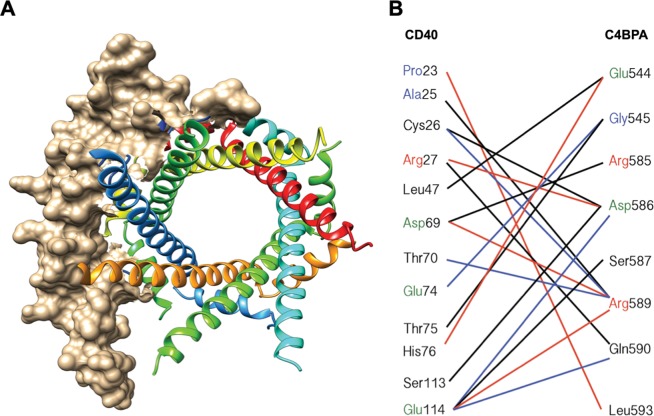
Structure of CD40 (PDB code, 3QD6) - heptameric C4BPA (PDB code, 4B0F) interaction interface. (**A**) Protein-protein docking was conducted through FireDock Server, followed by analysis with use of the Chimera program (UCSF). (**B**) Residues making direct contacts are linked by lines, which are alternatively color-coded simply for a convenient visualization. Positive residues, red; negative, green; hydrophobic, blue; hydrophilic, black.

The major isoform of C4BP, a plasma glycoprotein complex of 570 kDa, consists of seven subunits of C4BPA and one C4BPB. Structurally, heptameric C4BPA are oligomerized at C-terminus (Trp540–Leu597) that contains 14 cysteines forming seven intermolecular disulfide bridges to constitute the core complex^17^. The N-terminus of C4BPA is comprised of eight complement control domain proteins (CCPs)^17^ and the oligomerized C-terminal domain can act as an adjuvant to enhance immune response^18^, so we hypothesized that the C-terminus of C4BPA mediates its interaction with CD40. A report analyzing the crystallographic structures of CD40 and CD154 (CD40LG), a canonical CD40 ligand for CD40 activation, has demonstrated that hydrophilic and charge interactions play a key role in interaction of CD40LG and extracellular domain (Pro20–Leu261) of CD40^19^. This CD40 domain contains three cysteine-rich domains (CRDs). The interaction interface of CD40 with CD40LG can be divided into two areas, patches A (CRD3 and half CRD2) and patch B (CRD1 and half CRD2), and patch B interactions are highly homologous. We analyzed interactions between the published structures of C4BPA and CD40 (Protein Data Bank accession code, 4B0F and 3QD6, respectively) (Fig. 3A). We found that residues predicted to mediate interactions between C4BPA and CD40 are mostly hydrophilic and contact sites of CD40 that are predominantly located in patch B (Fig. 3B).

### C4BPA stimulates p100 processing and pro-labor mediators in human placenta

**Figure 4:**
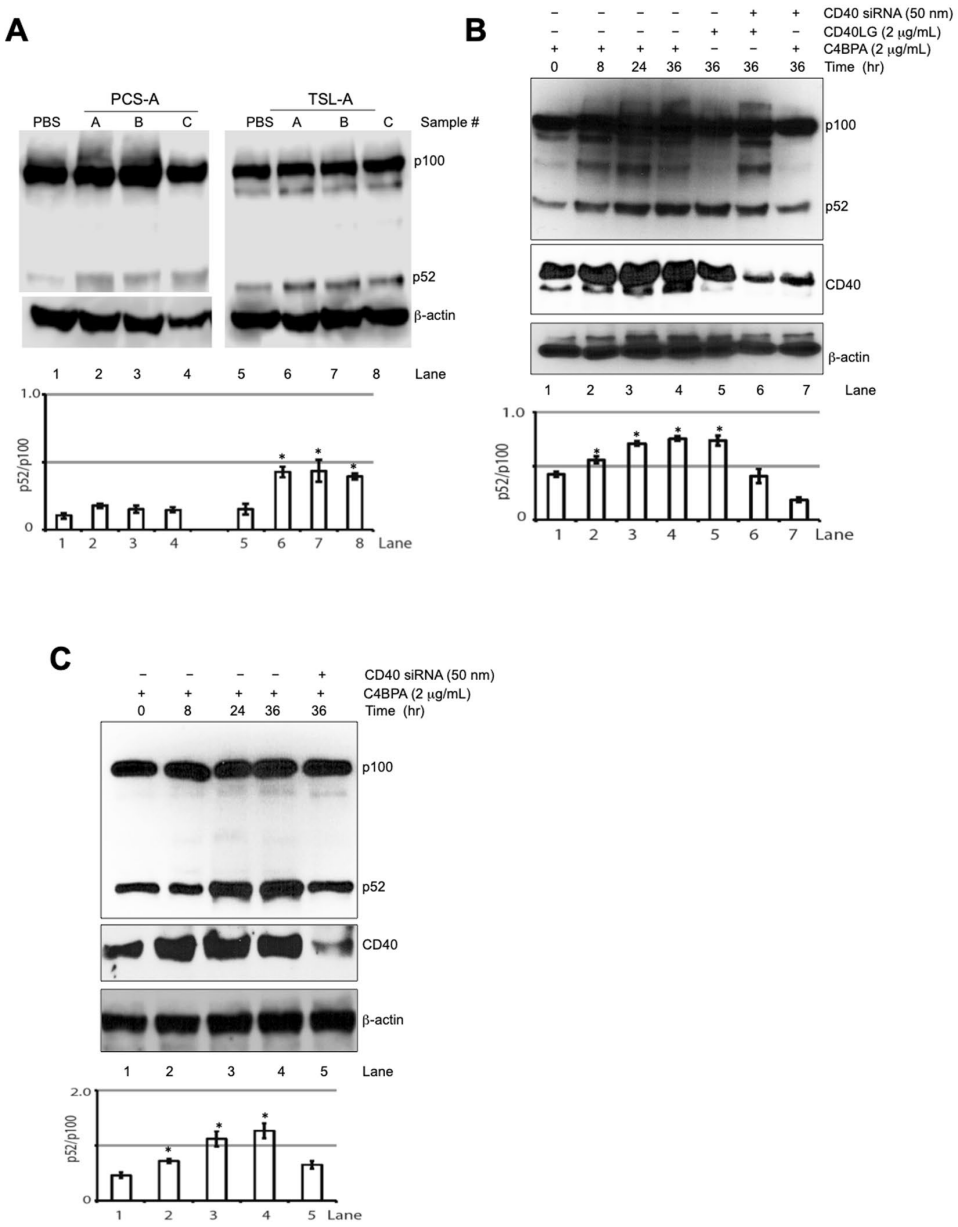
C4BPA activates non-canonical NF-κB signaling and pro-labor mediators in human placenta. (**A**) Term CTB were treated with the exosomes from fetal arterial umbilical cord blood at equal amount based on OD280, and protein extracts were analyzed by means of Western blot for the listed proteins. The graphs represent the ratio of p52 to p100, which were quantified with use of ImageJ program. The bars represent mean and standard deviation from experiments performed in 3 individual CTB samples by the same total exosome (exo) preparation (labeled with A, B, or C). (**B**) Term CTB were treated with C4BPA (Asn49–Leu597) or CD40LG, or in combination with CD40 siRNA transfection for indicated time period, protein extracts were analyzed by means of Western blot for the listed proteins. The bars represent mean and standard deviation from experiments performed in 3 individual CTB samples. (**C**) Term CTB were treated with C4BPA (Glu548–Leu597), or in combination with CD40 siRNA transfection for indicated time period, and protein extracts were analyzed by means of Western blot for the listed proteins. The bars represent mean and standard deviation from experiments performed in 3 individual CTB samples. *p < 0.01.

To investigate whether fetal signaling, and in particular, C4BPA is able to activate CD40 in CTB, and in turn drive non-canonical NF-κB activity, we first tested effects of the total exosomes purified from umbilical artery cord blood on processing of precursor NF-κB p100 protein to active p52, a molecular hallmark for activation of the non-canonical NF-κB pathway. CTB treated with the fetal exosomes led to an increased ratio of p52 to p100 (Fig. 4A, Supplementary Fig. 7). Treatment with the mature secretory form of C4BPA (Asn49–Leu597) promoted p100 processing in term CTB in a time-dependent manner, which was abrogated by treatment with CD40 siRNA (Fig. 4B, Supplementary Fig. 8). Of note, depletion of CD40 also led to inhibition of p100 processing stimulated by TCS-A exosomes (Supplementary Fig. 9). In addition, similar results were obtained when CTB was exposed to the C-terminal C4BPA (Gln548-Leu597) (Fig. 4C, Supplementary Fig. 10). In contrast, mutant C-terminal C4BPA (Asp586 to Arg, Gln590 to Phe, Leu593 to Arg) had negligible effects on p100 processing (Supplementary Fig. 11).

Collectively, we conclude that the C-terminus of C4BPA interacts with CD40 to activate non-canonical NF-κB signaling in the human placenta.

### C4BPA stimulates expression of pro-labor genes of human placental origin

**Figure 5:**
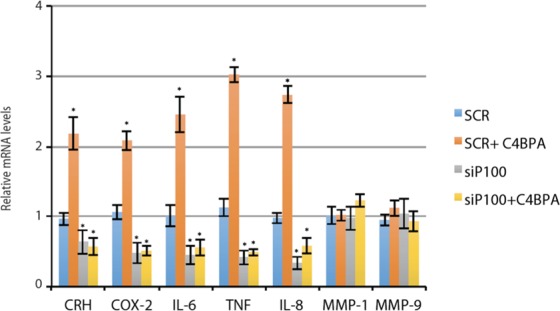
C4BPA induces expression of labor drivers in the human placenta. Term CTB were transfected with siRNA targeting p100 (siP100, which were in turn exposed to C4BPA for additional 24 hr. mRNA levels of CRH, COX-2, IL-6, TNF, IL-8, MMP-1, or MMP-9 were analyzed by means of RT-qPCR (N = 3 individual experiments). The bars represent average of relative mRNA levels and standard deviation from 3 individual experiments. *p < 0.01.

We have previously shown that the non-canonical NF-κB pathway drives *CRH* and *COX-2* gene expression and that p100 knockdown repressed these genes in term CTB^3^. In the current study, we assessed the effects of C4BPA and silencing of p100 on CRH, COX-2, and other proinflammatory mediators involved in the initiation of human labor. C4BPA drove expression of TNF, IL-1, IL-6, and IL-8 and these genes were suppressed by depletion of p100. MMP-1 and MMP-9 mRNA levels were not affected (Fig. 5).

These results further support that fetal C4BPA-mediated induction of labor drivers in the human placenta is dependent on p100 processing.

## Discussion

Taken together with our previous findings^4,5,20^, we hypothesize that the following mechanisms could contribute to the onset of human labor: First, as pregnancy progresses and the fetus grows and stresses the placenta, free glucocorticoid secreted by the fetus becomes available and drives p100 transcription^5^, which largely remains as a precursor form until C4BPA from the mature fetal lung exosomes stimulates CD40 to induce its processing to p52, thereby allowing RelB/p52 heterodimers to transport to the nucleus^20^. RelB/p52 heterodimers drive an epigenetic change at *CRH* and other pro-labor genes such as *COX-2*^4^, which may be a trigger for parturition.

Direct interaction of exosomes and target cells is mediated by ligand(s) and receptor(s) located on their respective surfaces, which are exemplified by exosomal fibronectin (FN1) and cell membrane-associated heparin sulfate proteoglycan glycoprotein (HSPG2), respectively^21^. Indeed, our study shows that FN1 was highly enriched in specimens of all PCS, TCS, and TSL groups (Supplementary Table 1). Combined with the data showing that HSPG2 is enriched in a variety of tissues including human placenta^15^, this suggests that FN1-HSPG2 interaction could play a role in anchoring of fetal exosomes to the surface of CTB.

Our study has revealed a novel fetal-placental signaling pathway that may contribute to the initiation of human term and preterm labor. A recent study has shown that maturation of the fetal lung and enhanced secretion of the surfactant components, surfactant protein A (SP-A) and PAF initiate a signaling cascade culminating in parturition in the mouse model^6^. Here we found that C4BPA produced by the fetal lung enters into the fetoplacental circulation to trigger activation of the non-canonical NF-κB pathway and drive pro-labor mediators in the placenta. The complement system and coagulation system have been long hypothesized to contribute to preterm labor^22^. To this end, McElroy and colleagues used VAAST^23^ to identify damaged genes and their disease-causing variants in maternal genome sequences from patients delivering preterm, and found 7 missense SNPs in *C4BPA* gene from 6 preterm birth exomes^24^. Combined with a previous study showing that multiple SNPs including rs11120211 were strongly associated with *C4BPA* expression^25^, this study further supports that a premature elevation of C4BPA secreted by fetal lung could play a role in the pathogenesis of preterm birth.

The results obtained from this *in vitro* study need to be further examined to determine whether these mechanisms would apply *in vivo*. Most animal models will not be suitable for these studies, which are exemplified by that CRH is not produced in mouse placenta^26^, and systemic progesterone decline is essential for activation of labor in mice^27^. Pregnancy in non-human primate models more closely resembles human pregnancy on the basis of multiple key physiological features, uterine anatomy, singleton gestation, and hemochorial placentation^28^. Indeed, genetic and pharmacologic manipulations of fetal-to-placental signaling pathways including C4BPA-CD40 will determine whether they are important determinants of the length of gestation in primates including humans.

## Methods

### Ethical approval

This study was approved by the Institutional Review Boards of Rutgers University (Pro20150001445). All patients signed a written informed consent for their specimen to be used for this study. All methods were performed in accordance with the relevant guidelines and regulations.

### Sample collection

Placental specimens from full-term pregnancies were collected from healthy women with estimated gestational age (EGA) of 37 and 40 weeks who were underwent Cesarean section (C-section). Women with complications of pregnancies, including diabetes, hypertension, autoimmune disease, infection (including HIV), fetal growth restriction, and preeclampsia, were excluded from the study.

Fetal cord arterial blood specimens were collected from healthy women in following 3 groups: (1) Patients having repeated C-section between 35 and 36 weeks of EGA after a previous classical C-section (PCS); (2) Patients following C-section at term with EGA between 39 to 40 weeks (TCS); and (3) Patients following spontaneous labor and vaginal delivery with EGA between 39 and 40 weeks EGA (TSL). In addition, fetal cord veinous blood specimens were collected from the same patients of TCS.

### Antibodies, siRNAs, reagents, and services

Anti-AFP, anti-APOH, and anti-C4BPA were purchased from Abcam (MA, USA). Anti-NFKB2 (p100/p52) antibody was obtained from Cell Signaling (MA, USA). Anti-E-cadherin monoclonal antibody was obtained from ThermoFisher Scientific.

FlexiTube siRNAs (4 siRNAs/per set) that have been functionally verified for silencing human NFKB2 (p100) and CD40 were obtained from Qiagen (CA, USA). Scramble siRNA with targeting sequence, 5′-AACAGUCGCGUUGUCGACUGGUU-3′, was synthesized by IDT DNA (IA, USA).

Recombinant C4BPA of mature secretory form (Asn49~Leu597) and C-terminus (Gln548-Leu597), and mutant C4BPA were purchased from Abcam (MA, USA), Creative Biomart (NY, USA), and GenScript (NJ, USA), respectively. We obtained CD40LG and power SYBR green PCR master (A25741) from ThermoFisher Scientific (USA).

Proteomics analysis of fetal exosomes was conducted at the Biological Mass Spectrometry Facility, Rutgers Robert Wood Johnson Medical School.

### Sequences of PCR primers

Primers (forward/reverse) for quantitative detection of mRNA include: CRH, 5′-GCAGTTAGCACAGCAAGCTCAC-3′/5′-CAAATGGCATAAGAGCAGCG-3′; COX-2, 5′-TGAGCATCTACGGTTTGCTG-3′/5′-TGCTTGTCTGGAACAACTGC-3′; MMP-9, 5′-AGGTGGACCGGATGTTCC-3′/5′-GGCACTGCAGGATGTCATAG-3′; MMP-1, 5′-TGGATCCAGGTTATCCCAAA-3′/5′-TGGAGAGTCAAAATTCTCTTCG-3′; IL-6,

5′-AGTGAGGAACAAGCCAGAGC-3′/5′-AAAGCTGCGCAGAATGAGAT-3′; IL-8, 5′-CTGCGCCAACACAGAAATTA-3′/5′-ATTGCATCTGGCAACCCTAC-3′; TNF, 5′-CCCCAGGGACCTCTCTCTAA-3′/5′-TCTCAGCTCCACGCCATT-3′; and GAPDH, 5′-CTCCCGCTTCGCTCTCTG-3′/5′-CTGGCGACGCAAAAGAAG-3′.

TCTCAGAATCTTCCTTTTGATGTG-3′/5′-CCAGTTGCAGATCCTACAAAGA-3′.

### Purification and culture of CTB

Purification of placental CTB was performed as recently detailed^3,29^. Briefly, villous tissue fragments containing no membranes received enzymatic digestions in a solution containing 0.25% trypsin, 0.2% DNase I, 25 mM HEPES, 2 mM CaCl_2_, and 0.8 mM MgSO_4_ in 1XHBSS at 37 °C. After three enzymatic digestions, cell pellets from the second and third digestions were pooled and resuspended in DMEM/F12 with 10%FBS. To further purify cytotrophoblasts, we used a discontinuous density gradient of Percoll (50%/45%/35%/30%) by centrifuging at 1000 g at room temperature for 20 min. Target cells at interface of fractions of 35%/45% were collected and further immunoprecipitated by an approach of negative selection with use of human CD9 and CD45 antibodies and Dynabeads (Invitrogen). Cells in the supernatant that were separated from Dynabeads with contaminated cells were pelleted, resuspended in DMEM/F12 plus 10% FBS (charcol stripped), plated at density of 2.5~3 × 10^6^/cm^2^, and maintained at 37 °C and 5%CO_2_ for at least 24 hr for syncytialization prior to further analysis.

### Isolation of fetal arterial and venous plasma exosomes

Fetal exosomes were isolated as previously established by differential ultracentrifugation^8^. Briefly, plasma was diluted with equal volume of PBS (pH 7.4) and exosomes were isolated through centrifugation was initially performed at 2,000 g for 30 min, followed by 12,000 g for 45 min to remove cell nuclei, mitochondria and debris. The supernatant was transferred to an ultracentrifuge polyallomer tube (ThermoFisher Scientific, USA) and was centrifuged at 200,000 g at 4 °C for 2 hr (Sorvall, S100-AT6, fixed angle ultracentrifuge rotor). The pellet was suspended in PBS (5 ml) and filtered through a 0.22 μm filter (Millipore, USA) to remove any microvesicles >220 nm in diameter. Then the filtrate was centrifuged at 200,000 g at 4 °C for 70 min, and re-spuspended in PBS for further analysis.

### Imaging of isolated exosomes by transmission electron microscope

Isolated exosomes (20 μL/per sample) were placed on a glow-discharged 400mesh for mvar/carbon coated grid. Excess solution was wicked off with filter paper. The grid was negatively stained with a solution of 1% uranyl acetate. The grid was imaged digitally on a Philips CM12 Transmission electron microscope at 80Kv attached with an AMT XR111 camera at Core Imaging Lab, RWJMS (Piscataway, NJ).

### *In vitro* exosome uptake assay

The total exosomes purified from 1 mL cord blood were resuspended in 100 μL PBS, and 10 μL exosomes were labeled with PKH67 Green Fluorescent Cell Linker Kit (Sigma-Aldrich, MO) according to the protocol provided by the manufacturer. Then PKH67-labeled exosomes were finally resuspended in 10 μL PBS and incubated with term human CTB in 90 μL culture media of DMEM/F12 for 24 hr with PKH67 only as the negative control.

### Immunofluorescence staining

Primary CTB treated with PKH67-labeled exsomes as described above were fixed with 4% paraformaldehyde in PBS and permeabilized for 10 minutes in 0.5% Triton X-100 in PBS at room temperature. Cells were then washed with PBS and incubated with anti-E-cadherin overnight at 4 °C. Cells were washed with PBS containing 0.1% BSA and incubated with fluorophore-conjugated secondary antibody (Alexa Fluor 532 goat anti-mouse, Invitrogen) for 1 hr at room temperature. The cells were counterstained with DAPI (Invitrogen) prior to being visualized under a fluorescence microscope (Nikon, Japan) according to the manufacturer’s instructions.

### High performance liquid chromatography/Mass spectrometry (HPLC/MS)

Proteomics analysis was performed at the Biological Mass Spectrometry Facility, Rutgers Robert Wood Johnson Medical School (Piscataway, NJ). Briefly, the exosomes were lysed in RIPA buffer (10 mM Tris-Cl (pH 8.0), 1 mM EDTA, 1% Triton X-100, 0.1% sodium deoxycholate, 0.1% SDS, 140 mM NaCl, and 1 mM PMSF) and boiled at 95 °C for 10 min. The supernatant was filtered with MWCO of 3 kDa (Millipore, USA). The filtrate were injected via an auto-sampler and loaded on Shimadzu C18 column (3 μm, 50 × 4.6 mm) at a flow rate of 0.5 mL/min using a Shimadzu HPLC system (Japan). The samples were separated using isobaric of 50% solvent A (0.1% Formic Acid) and 50% solvent B (Methanol). Samples were eluted, ionized by ESI, and detected by Shimadzu quadrupole mass spectrometer.

### Immunoprecipitation of exosomes

We coupled goat-anti-rabbit immunomagnetic beads (New England Biolabs, USA) coated with anti-AFP or C4BPA antibody by mixing them and rotating at 4 °C for 1 hr. The beads were washed three times with immunoprecipitation buffer (IP buffer; 10 mM Tris [pH 7.2], 10 mM MgCl_2_, 200 mM KCl, 0.1% Triton X-100). The antibody-bound beads were incubated with the total exosome at 4 °C for 1 hr with bidirectional mixing. The beads were washed four times (each wash of 5 min duration with bidirectional mixing) with IP buffer and twice with buffer containing 25 mM HEPES (pH 7.9), 10 mM magnesium acetate, 50 mM KCl, 1 mM EDTA, and 0.1% Triton X-100. The beads were collected for further analysis.

### Gene silencing

siRNA transfection was performed as previously detailed using transfection reagent Lipofectamine2000 (Invitrogen)^3,30^. Total RNAs were isolated from the cells and analyzed by RT-qPCR.

### Western blot

The samples were re-suspended in 1× SDS loading buffer, boiled at 95 °C for 5 min, and centrifuged. Supernatants were resolved on SDS–10% PAGE and transferred onto PVDF membranes (Bio-Rad). Membranes were blocked in 5% nonfat milk powder in PBST (10 mM phosphate buffer, pH 7.2; 150 mM NaCl; and 0.1% Tween-20) for 60 min, washed twice with PBST, and incubated with antibodies as indicated in 1% nonfat milk powder–PBST at 4 °C overnight. Membranes were washed three times with PBST, incubated with horseradish peroxidase-conjugated secondary antibodies at 1:5,000 in 1% nonfat milk powder–PBST, and developed by Immun-Star HRP Substrate (Bio-Rad, USA). The blots were visualized either by autoradiography or scanned by Chemiluminescence Western Blot Scanner (C-DiGit, LI-COR, NE, USA).

### Reverse transcription quantitative PCR (RT-qPCR)

Total RNAs were extracted by means of Trizol (ThermoFischer Scientific). Dried RNA pellets were re-suspended in appropriate volumes of DEPC H_2_O. The RNA was quantitated by OD_260/280_ using spectrophotometry and treated with DNase I (Promega, USA). Total cDNA synthesis was prepared by the oligo-dT primer method using the Superscript II Reverse Transcription kit (ThermoFischer Scientific).

The PCR program included denaturing (5 min, 95 °C), 40 cycles of melting (30 s, 95 °C), annealing (60 s, 55 °C), and elongation (60 s, 68 °C), and extension (2 min, 68 °C). PCR was performed using a StepOne Plus Real Time PCR System (Applied Biosystems) and power SYBR green PCR master mix (ThermoFisher Scientific).

### ELISA

Human PAF ELISA kit was purchased from ABclonal Science (MA, USA) and ELISA was performed according to the manufacturer’s protocol.

### Structural analysis of C4BPA-CD40 interaction interface

We first used the online STRING program, a functional protein association network^12^ to predict the interaction between C4BPA and CD40. Then, the docking of C4BPA (Protein Data Bank accession code, 4B0F) and CD40 (PDB code, 3QD6) was conducted through the online FireDock web server^31^, and the resultant PDB files were analyzed using Chimera (UCSF) software^32^ for predicting residues mediating interaction of CD40 and C4BPA.

### Statistical analysis

For proteomics analysis, the abundance of each protein was normalized to input samples. We used Student t-test to compare results of arterial blood samples of PCS and TCS, and paired t-test to compare arterial and vein blood samples of TCS because these two groups were obtained from the same patients. Because there were only three subjects in each group, permutation tests were further used for validating t-test results. Specifically, we used R-package “coin” to implement such tests. In the meanwhile, we used the Bonferroni correction to control the false positive rate at 5% for the three tests.

For other biochemical assays, each experiment was repeated a minimum of three times. Data (bars) are presented as mean ± standard deviation (SD). Student *t* test and one-way ANOVA were used to compare 2 and ≥3 groups subjected to attesting under a variety of conditions, respectively. p < 0.05 was considered statistically significant. If p < 0.05 in an ANOVA, a Dunnett’s test was used to compare the control with each test group.

## Supplementary information

Supplementary Figures and Table

## Data Availability

All data generated or analyzed during this study are included in this article and its Supplementary Information files.
