# Supplementary material for: Fetal lung C4BPA induces p100 processing in human placenta
Source: Sci Rep. 2019 Apr 2;9:5519. doi: 10.1038/s41598-019-42078-0 (PMC6445281; doi:10.1038/s41598-019-42078-0)
Supplement: Supplementary file 1 — Supplementary Figures and Table [file 41598_2019_42078_MOESM1_ESM.pdf]

Title

Fetal lung C4BPA induces p100 processing in human placenta

Mayra Cruz Ithier, [1] Nataliya Parobchak, [1] Stacy Yadava [1], Jerry Cheng [2], Bingbing Wang, [1*] and Todd Rosen[1*]

[1] Department of Obstetrics and Gynecology, Division of Maternal-Fetal Medicine, Rutgers Robert Wood Johnson Medical School, New Brunswick, NJ 08901, USA

[2] The Cardiovascular Institute of New Jersey, Rutgers Robert Wood Johnson Medical School, New Brunswick, NJ 08901, USA

* Corresponding author

Supplementary Figure 1

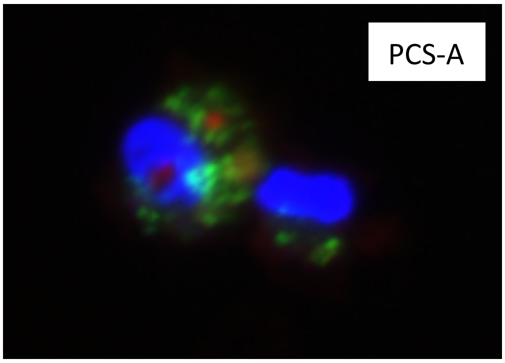

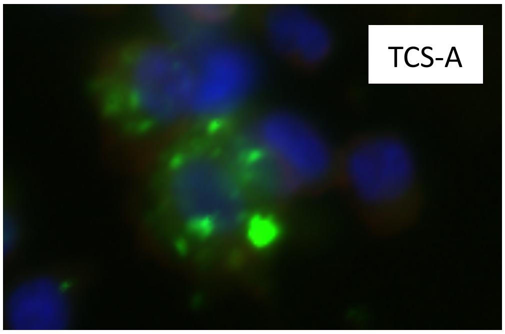

***In vitro* uptake assay.** Purified exosomes from were labeled with PKH67 followed by incubation with term human CTB for 24 hr as detailed in Methods. Green, exosomes. Red, E-cadherin. Blue, nucleus.

Supplementary Figure 2

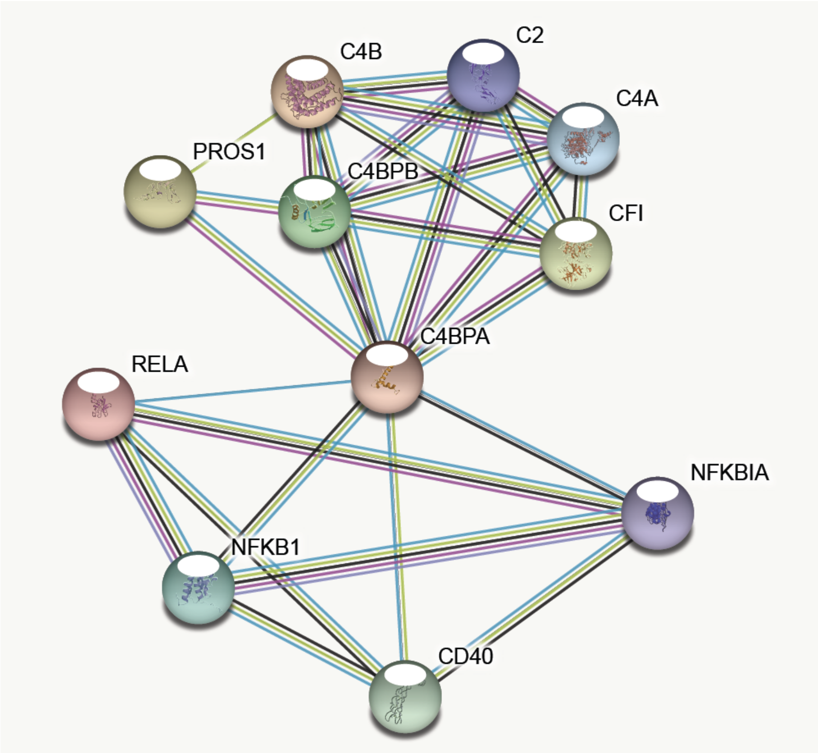

Interaction of C4BPA and CD40. We used STRING, a functional protein association network (https://string-db.org) to annotate and predict proteins interacting with C4BPA.

Supplementary Figure 3

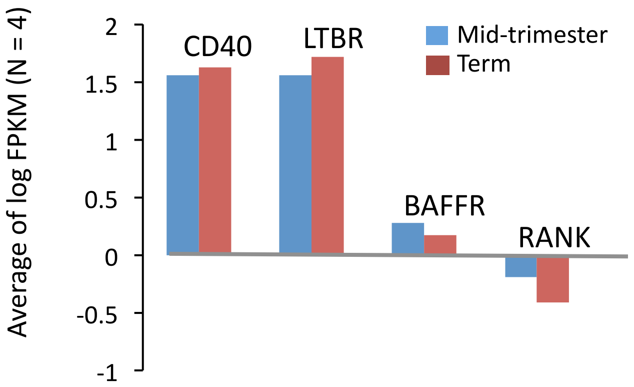

Expression of CD40 LTBR in CTB. Averages of log FPKM of genes as indicated by RNA-seq in human placentas at mid-trimester and term (N = 4 individual samples).

Supplementary Figure 4

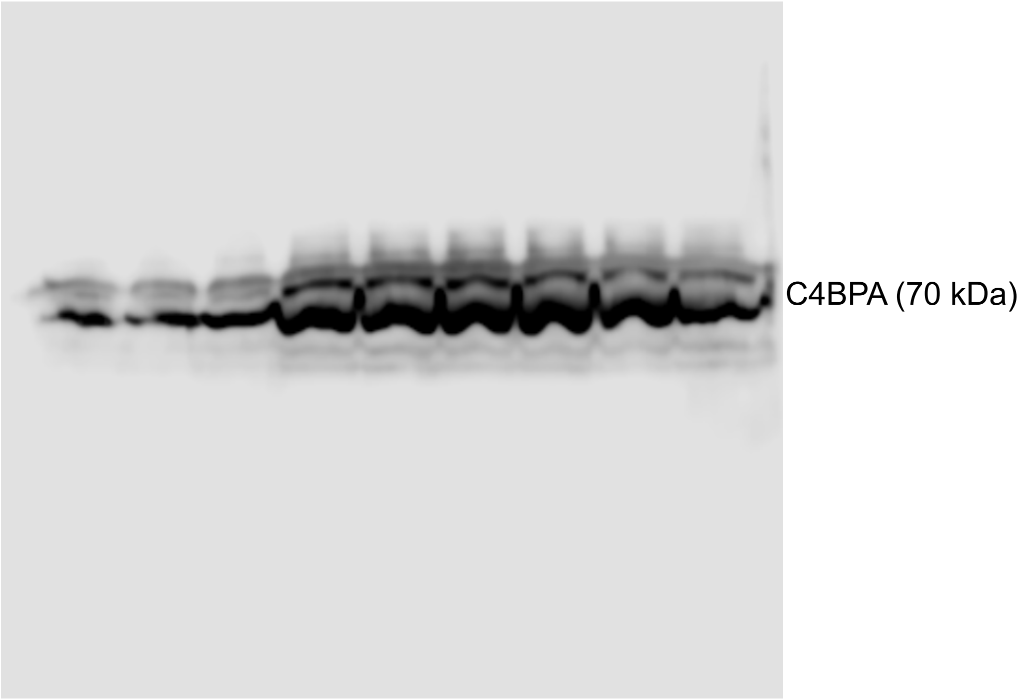

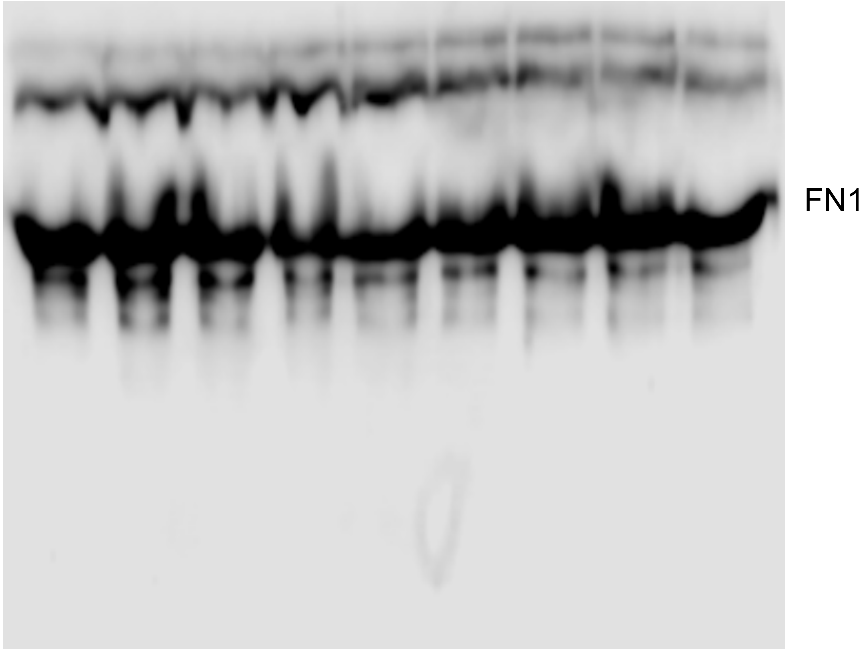

Full length blots for Fig. 1A

Supplementary Figure 5

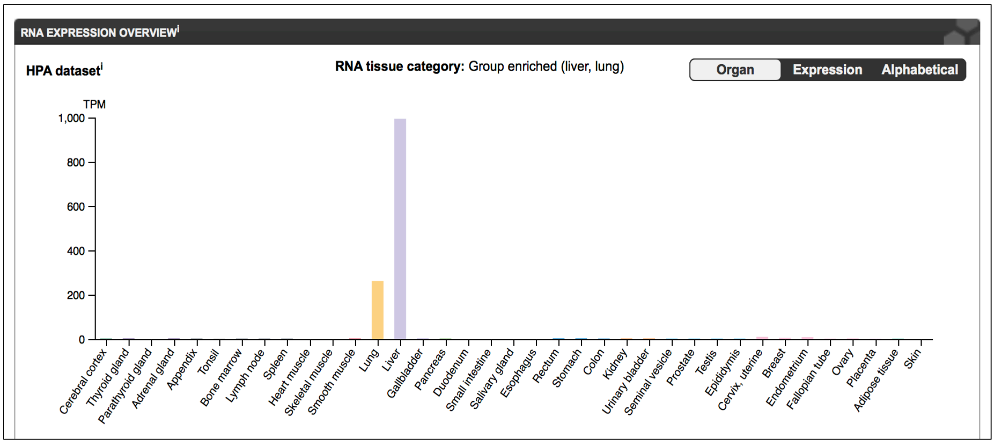

C4BPA

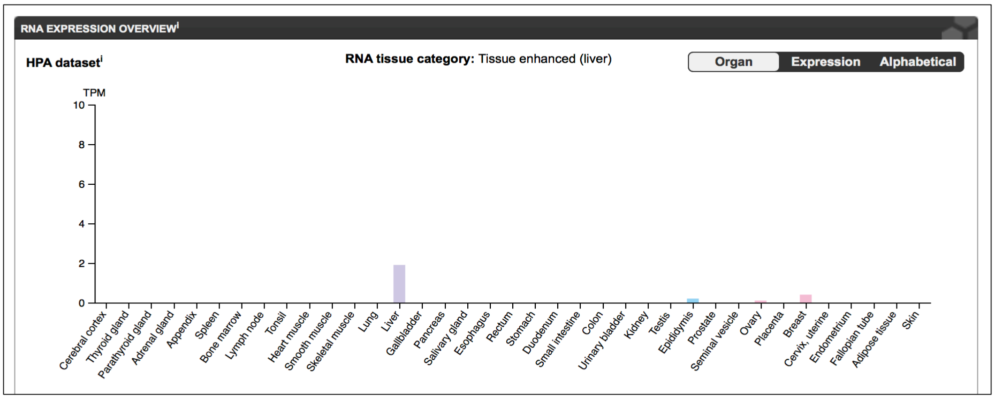

AFP

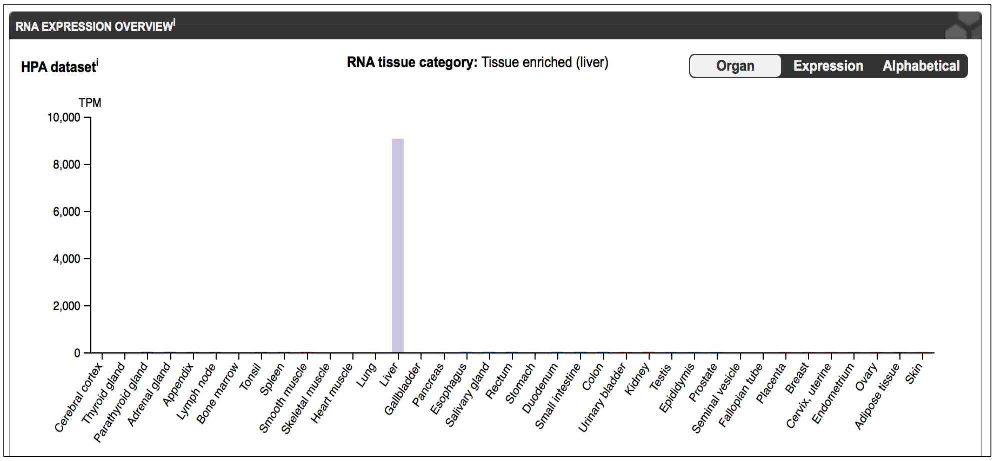

APOH

Tissue-specific gene expression of C4BPA, AFP or APOH. Snapshots from “The Human Protein Atlas” (http://www.proteinatlas.org). Orange column, lung; purple, liver.

Supplementary Figure 6

A

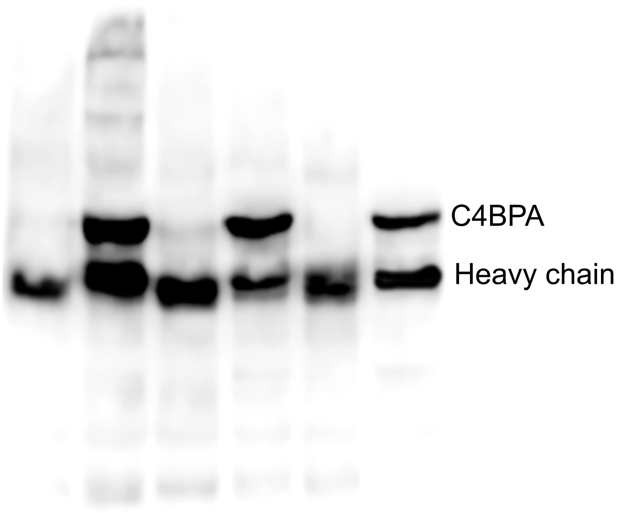

B

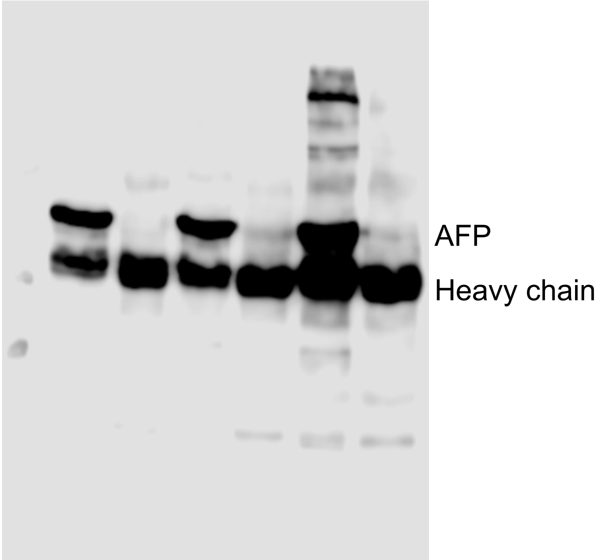

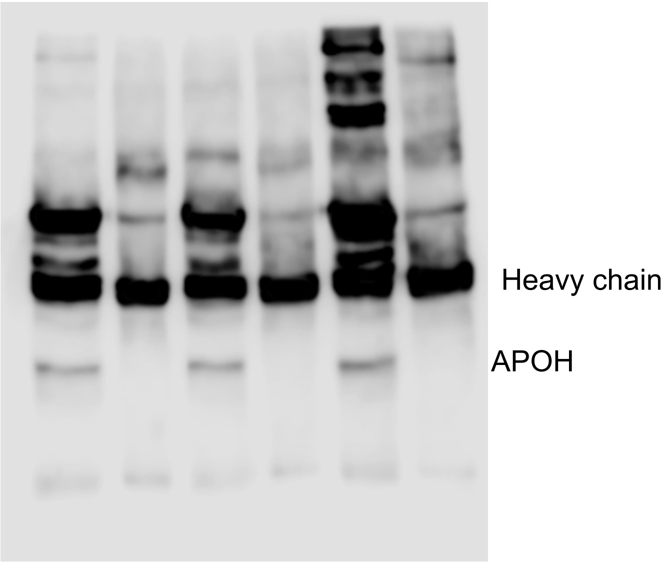

Full length blots for Fig. 1C

Supplementary Figure 7

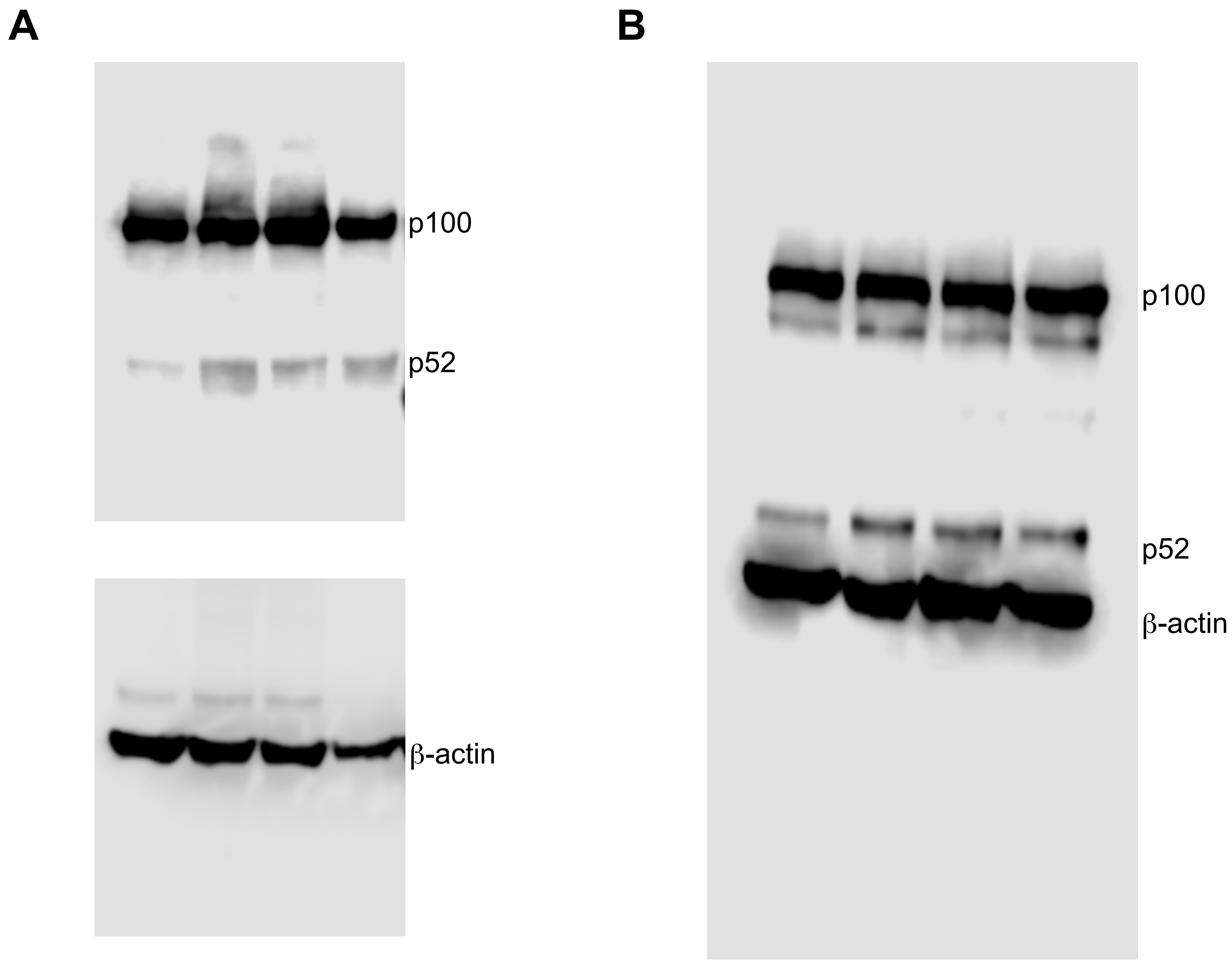

Full length blots for Fig. 4A

Supplementary Figure 8

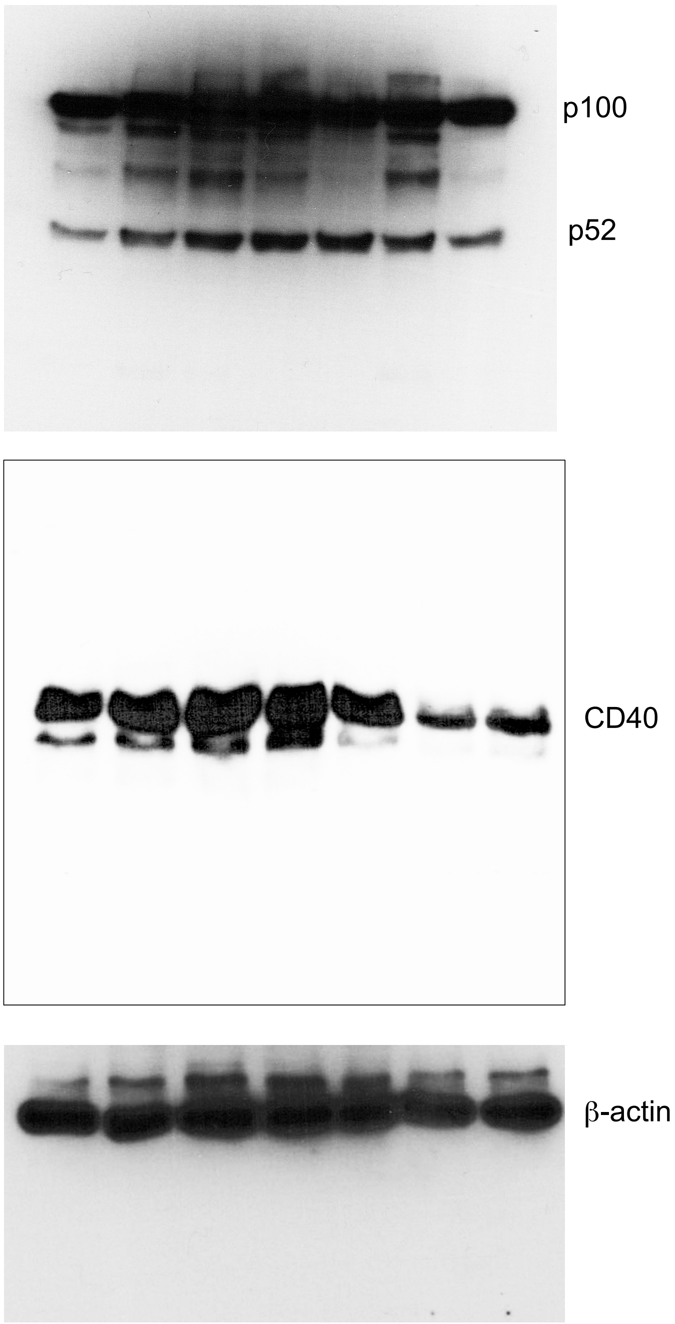

Full length blots for Fig. 4B

Supplementary Figure 9

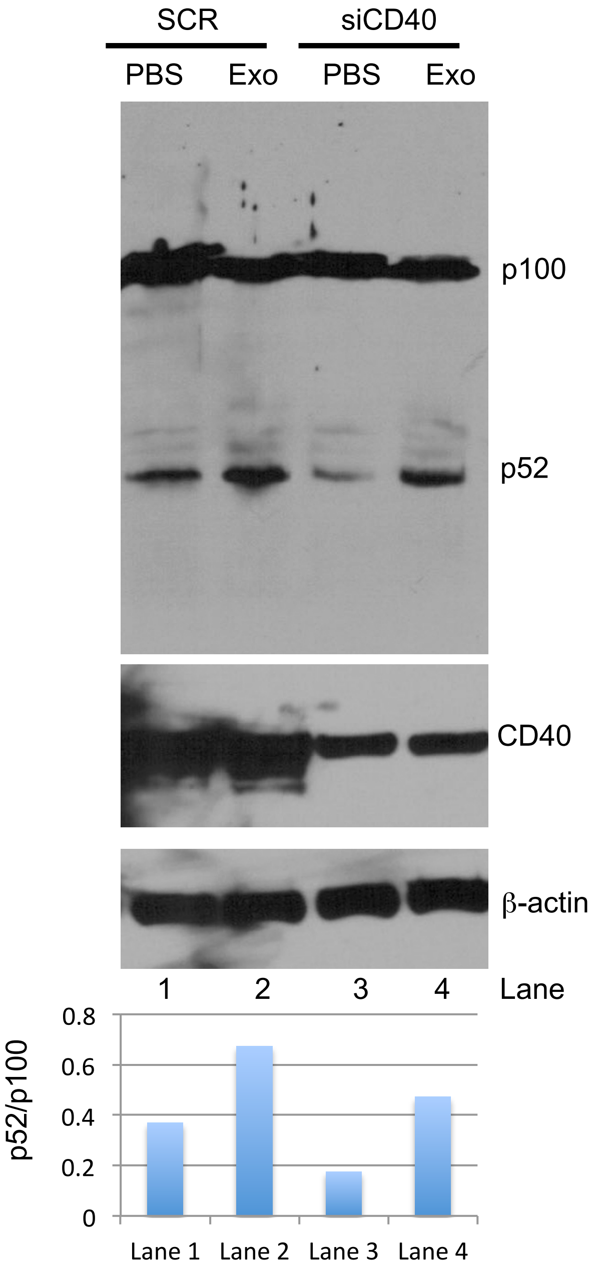

Depletion of CD40 led to Inhibition of exosomes-mediated p100 processing in term human CTB. Term human CTB were transfected with scramble siRNA (SCR) or CD40 siRNA (siCD40) at 50 nM for 24 hr, followed treated with TCS-A exosomes (exo) or PBS for additional 24 hr. The whole cell lysates were subjected to Western blot analysis.

Supplementary Figure 10

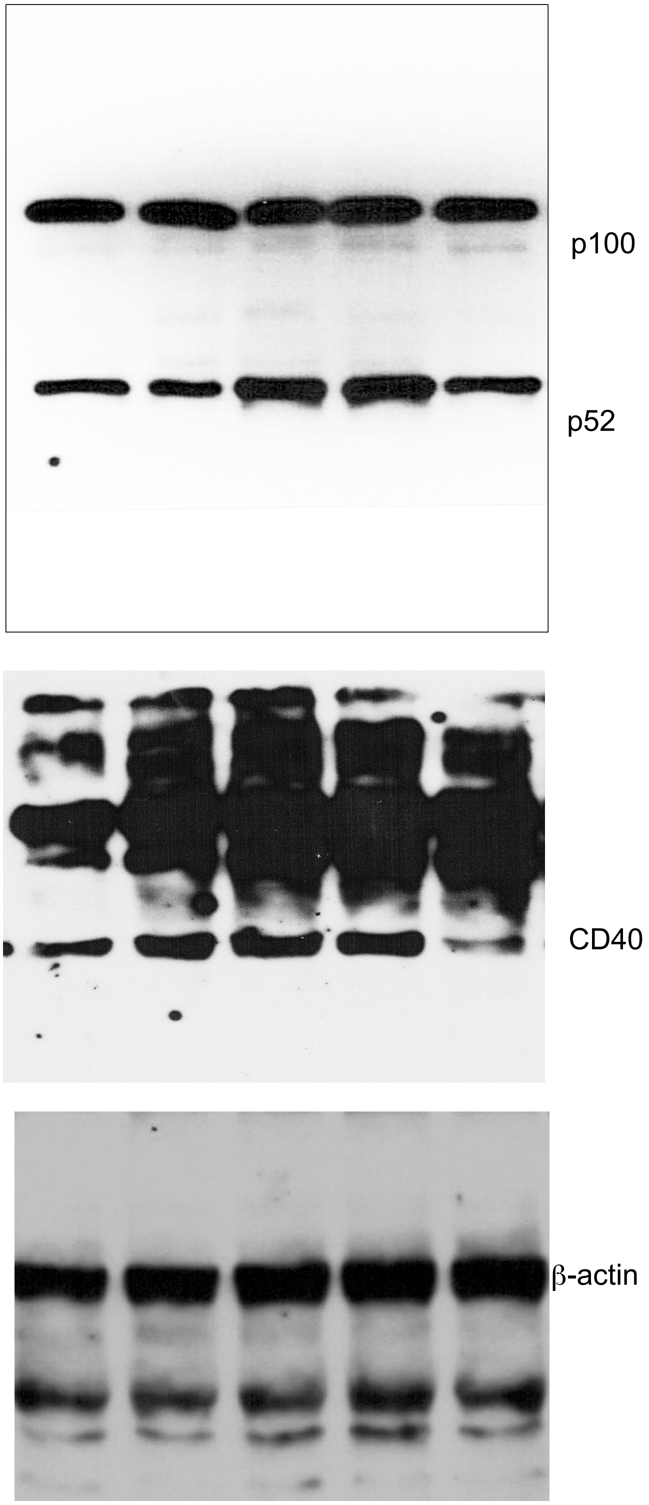

Full length blots for Fig. 4C

Supplementary Figure 11

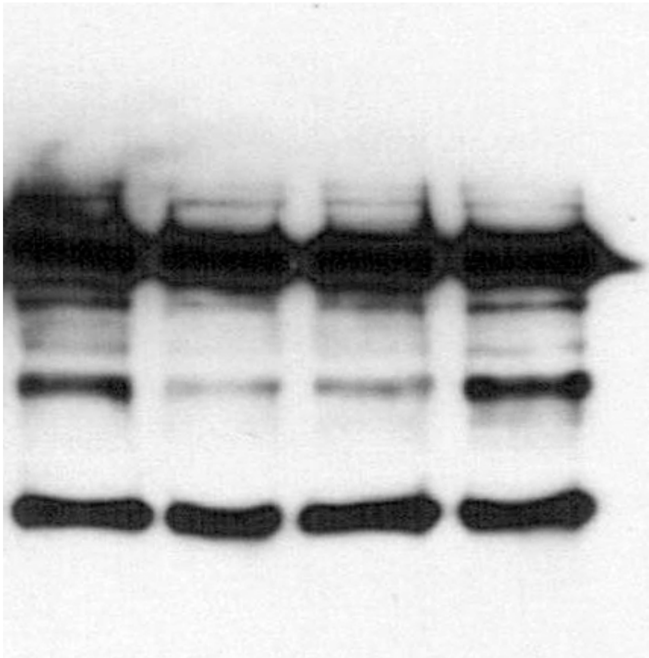

Mutant C-terminal C4BPA had on effects of p100 processing in term CTB. Mutant C4BPA (mC4BPA) included: Asp586 to Arg, Gln590 to Phe, Leu593 to Arg.

Supplementary Table 1. Proteomics analysis of fetal exosomes

Genes	PCS-A			TCS-A			TCS-V			TSL-A		
	A	B	C	A	B	C	A	B	C	A	B	C
A2M	2044	2015	2032	1982	2053	2154	1603	1469	1565	2088	2100	2172
ALB	1648	1545	1551	1442	1539	1597	2078	2143	2170	1139	1138	1209
C3	574	595	594	654	632	624	699	673	661	740	635	652
FGB	659	580	547	324	329	322	577	483	470	418	340	355
IGHG1	450	387	407	370	372	412	434	429	451	312	324	350
FGA	484	454	430	272	251	266	455	411	385	295	274	269
FGG	363	361	361	207	199	196	321	331	328	247	250	234
FN1	236	274	274	167	163	154	431	434	435	178	183	174
C4B	157	178	177	211	198	188	201	203	187	228	211	202
APOB	129	161	152	171	184	155	69	73	75	225	237	211
HBG2	195	192	185	229	209	233	60	43	33	176	154	175
IGKC	140	119	119	126	132	131	143	138	140	126	121	122
SERPINA1	152	138	131	128	137	129	151	156	146	97	86	87
HBA1	123	129	125	192	181	169	39	30	25	115	122	107
CFH	117	119	119	124	124	118	108	120	114	100	88	102
IGHG2	50	82	93	96	104	114	100	116	101	95	106	102
VWF	91	106	112	48	44	45	91	111	124	60	67	66
IGHG4	51	68	56	70	64	56	81	118	104	91	87	70
PLG	58	72	74	56	58	62	79	96	96	78	67	70
C5	58	78	76	62	70	65	69	83	84	75	68	70
CP	30	40	43	81	80	83	55	71	77	85	86	80
ITIH2	65	69	62	71	64	63	72	72	72	69	56	68
IGHG3	69	59	67	69	61	56	71	73	68	67	72	72
APOA1	110	70	69	79	79	59	95	59	64	47	38	46
IGHM	61	67	61	71	64	66	47	49	48	77	70	84
IGLL5	56	65	62	51	54	52	60	65	67	58	62	55
ITIH1	38	53	50	46	47	48	45	53	61	67	51	65
SPTA1	15	23	22	76	71	62	0	0	0	95	100	96
HBB	50	46	37	90	88	88	12	7	7	52	41	53
SLC4A1	24	29	31	57	59	58	1	2	1	83	108	89
SPTB	16	26	25	63	62	65	0	0	0	80	106	94
GC	43	39	39	43	44	42	55	66	61	39	41	39
ACTB	51	31	27	51	62	51	35	13	14	50	62	52
C4BPA	19	28	31	54	62	60	9	15	17	54	53	64
MYH9	64	29	28	57	55	51	37	14	10	39	56	41
SERPINC1	33	33	32	34	34	31	48	48	48	29	30	23
IGKV3-20	43	32	32	33	35	32	43	35	33	32	31	34
ITIH4	25	35	31	30	26	28	40	46	44	34	33	30
APOH	23	26	28	33	32	35	37	40	44	29	36	34
AFP	51	55	67	19	21	14	36	47	48	15	14	16
F2	30	34	29	27	30	28	41	44	40	31	26	28
ANK1	12	14	20	51	33	39	0	0	0	60	74	62
AHSG	31	31	29	29	28	29	45	49	48	20	20	19
GSN	28	36	35	19	22	22	32	44	39	32	29	23
CFB	26	29	31	28	29	27	26	37	33	28	34	31
VTN	23	25	26	32	26	28	20	29	24	37	32	38
C1R	11	18	19	29	33	30	21	25	18	37	39	37
APOE	24	25	22	28	27	29	25	26	25	31	30	30
IGKV1-27	36	27	29	28	31	22	43	22	27	23	19	20
LGALS3BP	32	23	24	29	25	24	26	24	26	26	29	27
IGHV3-21	28	27	28	27	27	22	23	21	20	29	24	24
FCN3	25	32	28	22	22	28	37	18	18	21	22	21
C1QB	27	29	26	25	28	21	24	23	23	26	20	20
SERPINA3	13	16	13	24	23	25	20	27	25	36	35	28
AFM	24	27	22	24	23	25	24	35	30	18	16	17
IGKV4-1	29	26	24	25	27	27	25	21	25	21	15	21
IGKV1-5	16	20	20	19	24	22	26	28	26	21	15	20
KNG1	22	19	19	20	24	19	27	28	29	18	16	16
TTR	25	20	17	21	24	17	37	28	24	18	15	12
HPX	19	16	20	18	20	19	26	30	29	19	16	13
CAT	22	30	26	29	27	27	2	1	1	26	25	24
A1BG	12	18	17	21	23	19	30	28	29	17	11	14
IGHV3-72	18	18	19	20	17	19	18	23	18	21	22	22
C7	22	25	18	21	18	13	26	26	28	11	11	15
CLU	18	17	17	19	19	16	13	21	19	24	18	21
FLNA	0	0	0	31	25	25	1	0	0	35	56	39
HBG1	29	23	21	28	30	30	9	4	6	23	8	18
IGKV3D-15	16	18	19	19	20	20	18	17	20	21	15	18
AGT	19	15	19	15	10	19	19	26	29	11	17	12
SERPING1	12	17	16	19	17	15	22	22	22	15	17	13
AMBP	15	18	17	19	13	17	17	21	24	15	15	17
C1QC	25	20	19	19	16	15	17	16	16	14	14	16
FCGBP	22	22	24	17	18	14	7	10	9	17	18	23
SERPINF2	13	16	16	11	12	13	24	24	27	14	15	13
TLN1	0	1	2	23	15	16	0	0	0	23	53	38
APOA4	17	19	17	18	13	11	17	22	15	9	13	9
GAPDH	13	8	10	24	21	21	0	0	1	29	23	23
IGHV4-59	16	17	21	12	15	11	19	14	17	16	6	12
TFRC	11	8	8	17	18	16	8	8	8	18	20	23
IGKV2-28	19	14	14	16	13	12	14	12	16	12	11	11
IGLV3-21	21	15	17	14	12	13	11	10	12	12	12	12
EPB42	3	3	6	21	19	15	0	0	0	23	32	26
C1S	5	10	10	13	10	7	14	15	12	16	18	14
THBS1	2	7	12	21	11	16	0	0	0	18	30	21
IGLV1-47	15	12	13	11	10	14	13	14	15	11	8	8

PON1	4	8	7	13	13	13	9	12	13	15	15	16
IGHV3-9	12	12	12	9	11	13	14	13	13	12	12	9
PGLYRP2	6	10	9	11	10	10	14	14	17	12	12	12
IGKV1-39	13	12	12	10	13	8	13	12	13	11	11	11
C6	10	12	12	11	11	10	10	13	15	10	11	12
KLKB1	5	9	8	13	13	11	10	11	13	9	14	16
IGLV3-25	11	14	12	9	12	9	9	10	10	13	8	10
C8B	3	10	10	8	7	7	7	13	10	15	17	12
IGHV5-51	11	10	13	14	10	11	13	9	10	10	6	9
FBLN1	6	10	12	7	9	8	6	12	15	10	13	10
SERPIND1	7	9	9	8	5	8	12	19	15	8	11	6
IGHV3-66	8	7	9	11	11	12	9	12	6	12	11	9
ECM1	12	10	12	4	7	6	16	17	22	5	3	3
PROS1	3	6	9	14	13	13	6	5	4	11	11	16
C1QA	4	15	11	6	8	7	7	9	9	10	13	10
CPN2	9	8	8	9	11	10	10	8	12	9	6	9
CFI	5	7	12	6	7	7	5	12	11	7	11	12
F13B	5	11	12	4	2	6	9	11	13	9	8	11
C4A	5	4	8	9	7	8	9	12	11	10	10	8
VCP	4	8	6	14	11	13	0	0	0	13	12	16
F12	5	5	8	9	8	10	8	12	9	7	8	10
F5	3	2	4	9	9	10	3	5	3	13	16	15
CD5L	8	8	11	10	8	7	3	8	8	7	7	8
ORM1	7	5	4	9	9	9	11	13	13	7	5	3
ITIH3	5	5	5	9	8	5	8	10	12	7	8	10
EPB41	0	1	1	11	10	12	0	0	0	14	20	19
ADIPOQ	3	9	6	9	8	7	8	9	9	8	8	7
SERPINA4	5	9	8	6	6	7	12	10	11	6	5	6
IGLV3-19	8	7	8	6	8	7	10	9	10	6	6	8
TUBA1B	1	2	1	11	14	13	0	1	1	14	14	14
MPO	0	0	0	11	13	10	0	0	0	13	16	19
RBP4	7	7	6	6	4	2	13	15	16	4	3	5
SERPINF1	5	7	9	6	6	7	9	12	10	4	4	5
ALDOA	7	6	5	11	12	15	6	2	1	9	7	6
KRT1	10	7	10	16	12	9	4	3	2	4	7	3
APOD	5	7	8	9	10	7	6	7	4	9	3	6
SERPINA7	3	6	6	5	5	7	8	13	13	4	5	6
IGKV1-12	4	7	7	6	4	6	6	8	10	8	7	9
C2	4	8	6	8	2	4	8	10	11	6	8	7
IGHV3-74	6	7	8	6	5	6	7	6	8	7	6	8
STOM	3	3	1	10	8	5	0	0	0	12	20	14
CPN1	2	5	7	8	6	5	7	7	6	7	8	10
PRDX2	6	8	6	11	10	8	2	1	1	10	8	8
CTSG	1	2	1	9	10	9	1	1	1	13	15	13
C8A	2	7	4	6	7	4	3	8	8	10	6	8
IGHV1-69	7	7	6	6	7	4	8	7	7	6	8	5
ATRN	4	5	6	8	7	6	7	7	5	5	6	8
SLC2A1	5	2	3	7	8	10	0	1	1	12	13	10
HRG	3	3	3	6	8	7	6	4	5	9	9	8
IGLV1-40	4	6	8	4	6	6	4	8	6	6	6	6
KRT10	5	6	8	19	6	8	1	4	2	2	6	3
IGHV3-33	8	5	6	7	6	3	7	7	4	4	7	5
APOA2	7	7	7	5	7	3	3	5	3	6	8	6
IGKV1D-33	4	7	7	4	7	7	4	4	4	6	5	3
LTF	0	1	1	9	7	8	1	0	1	11	13	10
PKM	0	0	0	10	12	8	0	0	0	9	11	12
LUM	1	6	6	4	4	4	9	10	10	4	3	2
CLTC	1	7	3	5	5	7	0	0	0	9	12	10
TGFBI	1	5	5	5	4	4	3	7	8	6	6	6
HP	2	3	4	5	4	6	3	4	4	6	7	10
ITGA2B	0	1	0	9	7	4	0	0	0	11	12	11
AZGP1	3	5	5	5	6	3	9	6	10	2	3	2
ITGB3	0	1	0	6	7	6	0	0	0	8	17	10
F13A1	4	3	4	4	4	4	6	4	6	5	8	5
CFHR1	4	3	4	5	4	4	7	8	8	4	2	4
TUBB4B	0	0	0	7	8	7	0	0	0	7	13	10
ENO1	0	1	1	6	8	7	0	0	0	10	9	9
LPA	8	13	13	1	2	1	2	1	2	2	4	2
IGKV1D-13	3	5	4	4	4	3	4	4	4	4	4	5
MST1	2	3	7	5	2	2	3	7	6	4	3	4
FCN2	2	3	3	9	7	8	3	4	2	2	3	3
TGM2	2	2	3	8	7	4	0	0	0	7	6	6
KRT9	4	3	8	14	3	3	2	1	1	2	7	1
HSPA8	2	2	3	8	8	7	0	0	0	7	5	5
PIGR	0	1	0	6	5	3	2	3	1	6	8	8
EEF1A1P5	1	1	1	6	4	6	2	0	0	6	10	5
HIST1H2BD	1	0	1	4	6	6	1	0	0	6	9	8
BCHE	4	3	3	4	3	1	4	3	3	4	4	4
ACTN1	0	0	0	5	3	1	0	0	0	7	11	9
CFP	4	3	7	1	1	3	3	6	3	2	3	2
VCL	0	0	0	4	4	2	0	0	0	6	11	8
HABP2	1	2	3	3	4	4	2	3	4	3	4	3
OLFM4	0	0	0	5	4	4	0	0	0	6	6	9
TMEM198	2	3	4	2	4	2	5	5	2	3	3	1
BLVRB	4	3	4	4	5	5	0	0	0	3	4	4
ORM2	1	1	2	2	2	4	4	6	5	2	3	2
FERMT3	0	0	0	3	2	4	0	0	0	7	10	7
IGHV3-43	3	2	3	4	4	4	3	3	2	2	3	4
APOC3	2	2	3	3	5	2	3	2	2	4	4	3

HBM	2	2	3	4	4	4	0	0	0	6	4	5
MASP1	1	5	6	1	4	1	4	4	3	1	3	2
RAP1B	0	1	1	4	4	5	0	0	0	6	6	6
LDHB	3	3	3	3	4	4	1	1	1	4	3	5
IGLV8-61	2	2	3	3	3	3	6	3	3	2	3	3
FASN	0	2	4	1	1	1	0	0	0	7	8	6
C9	0	1	1	4	3	5	1	3	3	4	4	3
IGHV3-15	3	3	4	2	1	2	2	1	3	4	3	4
CFL1	0	1	1	6	6	4	0	0	0	5	6	3
ACTC1	0	1	1	4	6	4	1	1	1	4	6	4
RAN	2	3	1	3	4	5	0	0	0	5	4	4
JCHAIN	3	3	3	3	3	3	3	2	2	2	2	3
CLEC3B	2	4	4	1	2	1	3	5	4	1	1	2
APOM	2	4	3	3	1	3	3	3	3	2	3	2
IGLL1	1	5	3	1	1	1	3	5	2	2	3	3
PZP	1	2	1	1	3	3	1	1	1	6	6	4
MBL2	5	3	3	5	5	4	0	1	1	2	2	2
IGHV1-2	1	1	1	1	1	1	1	3	3	2	10	3
IGLV3-1	2	3	3	2	2	2	2	3	3	2	3	3
IGHA1	0	1	3	7	1	1	2	4	3	2	3	3
PHGDH	7	2	3	3	2	3	4	1	2	2	1	2
GNAI2	0	0	0	3	2	3	0	0	0	6	8	5
C8G	2	1	2	3	2	4	3	3	3	3	1	2
SAA4	2	1	3	1	2	2	2	3	3	1	4	3
SERPINA5	1	1	3	3	0	3	4	3	3	2	3	2
HPR	0	1	2	1	1	2	1	4	3	4	4	3
GYPA	1	1	1	2	4	4	0	0	0	4	5	4
THBS4	0	3	2	1	1	1	0	3	0	4	4	5
TUBB1	0	0	0	4	2	3	0	0	0	2	8	5
APEH	0	1	1	4	4	3	0	0	0	4	4	4
IGLC7	2	3	3	2	1	1	3	4	4	1	1	1
IGHV3-73	1	4	3	2	4	2	0	1	1	3	1	2
IGLV4-69	2	2	2	3	2	3	3	1	3	2	1	2
ADD1	0	0	0	1	2	2	0	0	0	4	6	6
SELENOP	1	1	1	1	2	1	1	4	3	2	3	3
APCS	1	4	1	1	2	1	4	1	1	3	1	3
IGHV2-5	1	5	4	1	1	1	2	3	3	1	1	1
IGKV6D-21	2	1	1	2	3	1	0	3	4	1	2	3
MYH10	5	2	2	2	3	2	2	1	1	0	4	1
PSMB5	2	4	2	3	1	2	0	0	0	2	5	2
PPIA	1	2	1	2	4	2	0	0	0	4	4	3
EPX	0	0	0	3	3	3	0	0	0	4	5	5
SELENBP1	2	3	3	2	1	2	0	0	0	4	3	3
FLOT2	0	0	1	4	4	3	0	0	0	4	4	4
PSMB6	1	2	3	2	2	4	0	0	0	3	3	2
IGLV3-27	1	3	4	1	1	2	3	3	3	2	0	1
YWHAZ	0	0	1	4	4	3	0	0	0	4	3	4
ACLY	0	2	3	4	4	1	0	0	0	3	3	2
ITGAM	0	0	0	2	1	2	0	0	0	4	6	5
CAPN1	0	1	2	4	4	1	0	0	0	3	3	4
ALAD	1	2	1	4	3	2	0	0	0	3	1	5
MSN	0	0	0	2	2	2	0	0	0	4	6	4
LBP	1	0	1	1	1	1	0	2	0	3	4	5
SERPINA6	1	1	1	1	1	1	4	5	3	1	1	1
PSMA4	3	1	3	3	1	1	0	0	0	4	2	3
IGFALS	1	0	2	2	1	2	3	4	4	1	1	1
MYL6	2	1	1	2	4	4	2	0	0	1	4	2
PSMA3	1	2	3	4	2	2	0	1	1	1	2	2
APOC2	1	2	2	1	3	1	2	1	1	2	3	2
IGKV1-16	2	2	3	1	1	1	2	1	2	2	1	2
F10	3	2	1	4	1	1	3	2	3	0	1	2
IGHV6-1	1	1	2	1	1	1	2	1	3	2	3	2
PRTN3	0	0	0	2	3	4	0	0	0	3	4	4
S100A8	0	1	1	4	3	4	0	0	0	3	3	2
PRDX6	1	2	1	2	4	2	0	0	0	1	3	3
CD36	0	0	0	3	1	1	0	0	0	4	6	3
TKT	0	0	0	3	1	2	0	0	0	4	4	3
HSP90AA1	0	0	0	4	4	1	0	0	0	2	4	3
ABCB1	2	2	1	1	3	2	0	3	1	1	3	1
APMAP	0	1	1	3	3	2	1	0	0	2	3	2
CCT3	0	0	0	3	2	1	0	0	0	3	5	3
PSMB2	0	1	2	1	0	1	0	0	0	4	4	5
CORO1A	0	0	0	1	1	1	0	0	0	4	4	5
IGHV1-24	1	2	2	1	0	1	3	2	3	1	2	1
HBZ	2	2	2	3	2	1	1	1	1	1	1	2
IGKV3D-20	1	2	1	3	1	1	3	1	2	1	0	2
S100A9	0	0	0	2	1	2	0	0	0	4	4	4
FLOT1	0	0	0	0	1	1	0	0	0	4	6	5
PSMB1	0	1	1	2	1	1	0	0	0	2	2	5
DEFA1	0	0	0	3	3	2	0	0	0	4	3	2
GPLD1	0	1	1	1	1	1	2	2	1	2	3	2
PLTP	1	2	1	1	1	0	1	3	2	2	2	1
HSPA5	0	0	1	2	2	1	0	0	0	2	3	4
WDR1	0	0	0	1	2	1	0	0	0	2	5	4
ANPEP	2	5	3	1	1	0	1	1	1	1	1	2
UBA52	2	1	1	1	3	1	1	0	1	1	1	3
ADD2	0	0	0	2	2	1	0	0	0	2	4	3
PSMA6	0	1	1	1	1	1	0	0	0	1	5	3
CCT7	0	0	0	1	2	1	0	0	0	2	2	6

PSMA5	1	2	1	2	2	1	0	0	0	2	2	1
C4BPB	1	1	0	1	1	1	0	1	1	4	2	2
PF4	0	1	2	1	2	3	0	0	0	2	1	2
PSMA7	1	1	1	2	2	1	0	0	0	2	1	2
ANXA6	0	0	0	1	3	2	0	0	0	2	4	3
IGLV4-60	2	1	1	1	1	2	1	2	1	1	1	2
LRG1	0	1	1	1	2	0	3	4	2	1	1	1
EFEMP1	0	1	1	1	1	0	0	3	3	1	1	2
PSMA1	0	1	1	4	2	1	0	0	0	2	1	2
DMTN	0	0	0	1	1	1	0	0	0	3	4	3
PSMB4	0	0	2	1	2	1	0	0	0	3	4	1
APOC1	0	1	1	1	1	2	0	1	1	1	3	2
IGHV4-39	1	1	2	1	1	0	1	1	1	2	2	2
APOL1	1	1	0	1	1	1	3	1	2	1	2	1
ITGB2	0	0	0	1	2	2	0	0	0	1	4	3
PFKL	1	1	0	2	0	1	0	0	0	1	4	3
CFHR2	0	1	1	1	1	1	1	3	2	1	1	1
PSMA2	0	1	1	2	2	1	0	0	0	1	2	2
CCT6A	0	0	0	1	0	1	0	0	0	2	4	4
MPP1	0	0	0	2	1	1	0	0	0	1	4	3
PLEK	0	0	0	1	1	1	0	0	0	2	3	4
PFN1	0	0	0	3	1	2	0	0	0	2	2	3
POSTN	0	3	2	0	0	0	0	3	3	0	1	1
PRPS1	0	0	0	3	1	0	0	0	0	2	3	3
GP1BA	0	0	0	1	0	1	0	0	0	2	4	3
COLEC11	1	1	1	1	1	1	0	1	1	1	3	1
YWHAE	0	1	0	1	1	2	0	0	0	2	2	2
RHOA	0	0	0	1	1	1	0	0	0	3	2	3
GNB1	0	1	0	3	2	0	0	0	0	1	3	2
PI16	0	1	2	1	0	1	0	2	3	1	1	1
C18orf63	1	1	1	1	1	1	1	1	1	1	1	1
GYPC	1	1	1	1	1	1	0	0	0	2	2	2
CPB2	0	0	1	1	1	0	2	1	2	1	1	1
CAMP	0	0	0	0	1	2	0	0	1	2	3	2
GPI	0	0	0	1	1	1	0	0	0	2	3	2
SPP2	0	1	1	0	0	0	1	3	3	0	1	1
CCT4	0	0	0	1	1	1	0	0	0	3	2	2
ANXA7	0	0	0	3	4	3	0	0	0	1	1	0
TPI1	0	0	1	3	2	3	0	0	0	1	1	1
LCP1	0	1	0	1	1	0	0	0	0	2	3	2
CFD	0	1	1	0	0	1	2	3	3	0	0	1
NME1	0	1	1	1	2	1	0	0	0	1	2	1
CD9	0	0	0	1	1	2	0	0	0	2	3	1
C1RL	0	1	0	1	0	0	2	1	2	0	2	1
PAICS	0	1	0	1	0	0	0	0	0	2	3	3
MMRN1	0	1	1	0	1	0	1	1	0	1	2	2
ILK	0	0	0	1	1	1	0	0	0	1	2	3
MEP1A	4	3	4	0	0	0	0	0	0	0	0	0
ATP5B	1	0	0	1	0	0	1	0	0	1	3	3
GP1BB	0	0	0	0	2	1	0	0	1	2	2	2
CETP	0	0	2	0	0	0	0	1	1	1	3	1
RAC2	0	0	0	1	1	1	0	0	0	2	2	3
TUBB	0	0	0	2	1	1	0	0	0	2	3	1
CA1	0	0	0	2	4	1	0	0	0	1	0	1
HSPD1	0	0	0	2	2	0	0	0	0	1	3	2
HBD	0	1	0	2	2	1	0	0	0	2	1	2
LYZ	2	1	1	1	1	1	2	1	0	1	1	0
GPX3	0	1	1	1	1	1	1	1	1	1	0	0
SHANK1	0	1	1	1	0	1	0	3	2	1	1	0
ACTR3	0	0	0	1	2	1	0	0	0	1	3	2
ATP5A1	0	0	0	1	1	0	0	0	0	2	3	2
CAP1	0	0	0	1	0	0	0	0	0	2	3	2
XPO7	0	0	0	1	0	0	0	0	0	2	3	2
CD44	0	0	0	1	1	1	0	0	0	2	2	1
CAVIN2	0	0	0	1	1	1	0	0	0	1	4	2

All counts were normalied to total inputs.
